# Morphological Analysis of KDP-Crystal Workpiece Surfaces Machined by Ultra-Precision Fly Cutting

**DOI:** 10.3390/ma13020432

**Published:** 2020-01-16

**Authors:** Dongju Chen, Shiwei Zhang, Jingfang Liu, Chunqing Zha, Ri Pan

**Affiliations:** Beijing Key Laboratory of Advanced Manufacturing Technology, Beijing University of Technology, Beijing 100124, China; djchen@bjut.edu.cn (D.C.); zhangshiwei@emails.bjut.edu.cn (S.Z.); chachunqing@163.com (C.Z.); panri@bjut.edu.cn (R.P.)

**Keywords:** potassium dihydrogen phosphate (KDP) crystal, surface morphology, fractal feature, power spectral density

## Abstract

Potassium dihydrogen phosphate crystals exhibit excellent nonlinear optical properties that are significantly affected by the surface morphology of the crystal. To comprehensively examine and characterize the morphological features of these crystals, the machined surfaces of workpieces are analyzed using wavelet, fractal, and power spectral density (PSD) methods. First, the fractal method is employed to analyze the features of the machined surfaces of different materials and examine the relationship between the surface roughness and fractal dimension of different materials. Then, the morphological anisotropy of the machined surfaces is analyzed using the two-dimensional PSD method. Based on the orientation of the machined surfaces of the workpieces, the tangential waves on the surfaces are analyzed using wavelet-transform and PSD methods. From a frequency-domain perspective, the scales of various influencing factor signals are identified. Additionally, the frequency range of the spindle vibration is determined based on the machining experiment. On this basis, the cause of the machined surface waviness errors is revealed.

## 1. Introduction

Potassium dihydrogen phosphate (KDP) crystals are a type of nonlinear optical and electro-optical crystal material with exceptional properties. Due to the excellent nonlinear optical properties of these materials, KDP crystals are extensively applied in the components of frequency multipliers, optical modulators, three-dimensional (3D) optical data storage devices, and new-generation 100-joule laser systems [1]. In recent years, the development of solid-state laser technology has led to stricter quality requirements for the machined surfaces of KDP crystals. The surface performance metrics of KDP crystals mainly include form, waviness, and roughness errors [2]. Among them, the waviness error will affect the use of mechanical parts, so the impact of surface waviness has caused many scholars to study this in depth. Adamczak et al. [3] found that an increase in the surface waviness on the inner and outer raceways causes an increase in the vibration level. This effect is most visible for the medium vibration frequency range. Costa et al. [4] addresses the conception of a measurement system to objectively and systematically assess paper superficial waviness in industrial practice. Cao et al. [5] studied the surface waviness of woven ceramic matrix composites and found that surface waviness exerts a tremendous influence on use performance and working life, and the influence degree is dependent on the particular type of surface waviness. For surfaces of high-precision optical workpieces machined by ultra-precision fly cutting, the waviness error of the workpiece surface may cause laser damage to the optical components and directly affect its performance [6]. Therefore, to further improve the precision of the machined surfaces of workpieces, waviness errors are an important factor that cannot be overlooked.

Many factors will affect the morphological characteristics of the workpiece, such as mechanical vibration [7], crystal orientation [8], material physical properties [9], and spindle speed [10]. Chi et al. [11] studied the effects of blade tip vibrations, material properties, and spindle rotation errors on ultra-precision diamond-turned surfaces through multispectral analysis. Chen et al. [12] determined the effects of the frequency response of the spindle on frequency-domain errors of machined surfaces by analyzing aerostatic spindle errors. An et al. [13] examined the effects of an unbalanced aerostatic spindle on the machined surfaces of workpieces and found that the waviness errors caused by the unbalanced spindle were less than 0.1 µm and the wavelength was approximately 100 nm. Based on the kinematic principle of planar milling, Baek et al. [14] established a surface roughness simulation model and used the model to study the effects of radial runout errors and feed speed on machined surfaces; these researchers found a strong nonlinear relationship between the feed speed and surface roughness. Through experiments on diamond turning of an aluminum alloy (Al 6061) and a single-crystal Al, Cheung et al. [15] studied the effects of the material properties on the formation of the surface microstructure of the workpieces. However, these studies primarily focused on the effects of various factors on the surface morphology, and there is a lack of systematic research on the surface morphology of KDP crystals.

To comprehensively analyze and characterize the morphology of the machined surfaces of workpieces, the machined surfaces of the KDP crystal are analyzed using wavelet, fractal, and power spectral density (PSD) methods. First, the fractal method is used to determine the 3D fractal dimension (*Ds*) of the machined surfaces of various materials. Then, the surface roughness–*Ds* relationship is analyzed for each material. Additionally, the fly-cutting-machined KDP crystal surface is found to exhibit high anisotropy using the two-dimensional (2D) PSD method. Based on the orientation of the machined surfaces of the workpieces, the tangential waves on the surfaces are analyzed using the wavelet-transform method. From a frequency-domain perspective, the scale of each influencing factor signal is identified. The PSDs are analyzed and compared with the simulated values. On this basis, the frequency range of the spindle vibrations is identified. Furthermore, the cause of the machined surface waviness errors is revealed.

## 2. The Structure of KDP Crystal and the Analysis Method of Surface Morphology

### 2.1. The Structure of KDP Crystal and Crystalline Orientation

**Figure 1:**
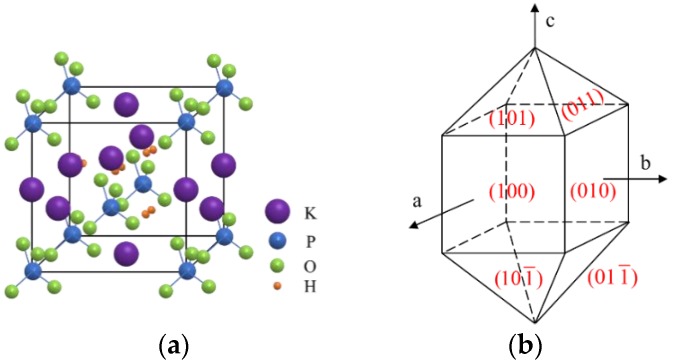
The structure of a KDP (potassium dihydrogen phosphate) crystal: (**a**) The cell structure of a KDP crystal; (**b**) The ideal shape of a KDP crystal.

KDP crystals belong to a tetragonal crystal system at room temperature, with the D_2d_-42m point group and the D^12^_2d_-I42d space group. The unit cell parameters are a = b = 0.7453 nm, c = 0.6975 nm, and Z = 4. The cell structure of the KDP crystal is shown in Figure 1a. Its ideal shape is one formed by the aggregation of a quadrangular column and a quadrangular double cone, as shown in Figure 1b. The structure of the KDP crystal determines its properties. A KDP crystal is an anisotropic material with strong anisotropy in physical and mechanical properties.

**Figure 2:**
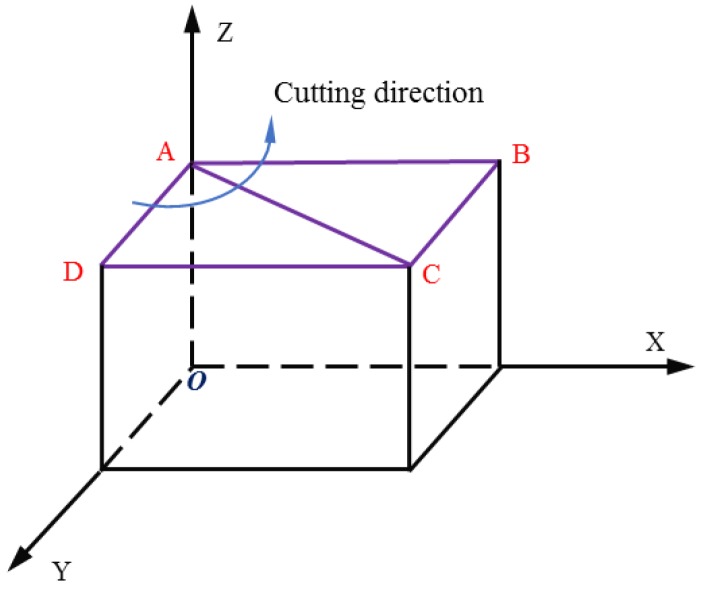
The crystalline orientation of the KDP crystal.

A schematic diagram of the crystal orientation of the KDP crystal is shown in Figure 2. The plane ABCD is the (001) crystal plane. The directions of AD, AC and AB are the crystalline directions of [100], [110] and [010], respectively. The crystalline directions of [100], [110] and [010] are defined as 0, 45 and 90 crystalline orientation, respectively.

### 2.2. Morphological Orientation of Surfaces

If the physical properties of the material of a workpiece are consistent in various directions, then the physical properties of the material are unrelated to the orientation, and the material is isotropic; otherwise, the material is anisotropic. Like the physical properties of the workpiece material, the topological structure of the workpiece surface is also isotropic and anisotropic. The isotropic workpiece surface has the same topological structure in all directions, while the anisotropic workpiece surface may have a different topological structure in different directions.

Based on the properties of surfaces, Thomas categorized anisotropic surfaces into weakly and strongly anisotropic surfaces [16]. The profile information of a weakly anisotropic workpiece surface is different in each direction, and the profile information in any direction plays a major role on the entire surface of the workpiece. For a strongly anisotropic workpiece surface, the profile information of only one direction plays a major role for the entire surface of the material, whereas the profile information of all the other directions can be treated as the superposition of the profile information in this direction along the orthogonal direction. Therefore, when analyzing strongly anisotropic machined surfaces, it can be simplified by analyzing the surface profile information in one direction. *Ds* can comprehensively reflect the surface topology of a workpiece and thus can be used to study the orientation of the machined surface of a workpiece.

### 2.3. Fundamental Theory of the Fractal Method

On a microscopic scale, the surface of the workpiece is rough and not smooth. Surface profiling shows that on a microscopic scale, as magnification increases, a surface profile does not tend to be a straight line but instead maintains an approximately similar shape. The state where a part of an object is similar to the whole in a certain way is called fractal, and the fractal geometry has self-similarity and scale invariance [17]. The fundamental principle of the fractal method is as follows,

(1) $$D=\frac{\ln N}{\ln s}$$

where *D* is the theoretical fractal dimension and *N* and *s* are scale parameters related to self-similar features.

### 2.4. Calculation of Ds

*Ds* is a minor modification of the conventional concept of dimensions. This modification allows the concept of non-integer dimensions. *Ds* is the most important characterization parameter in fractal theory. This parameter reflects the microscopic morphological features of a surface. The more complex the microscopic morphology is, the greater the *Ds* is. Box counting is a method commonly used to calculate *D_s_*. This method can be used to perform calculations on curves or areas encircled by curves, e.g., surface morphology, pore distribution, and crack propagation. Box-counting dimension can be represented by the following,

(2) $$D_{s}=\underset{k\to \infty }{\lim}\frac{\ln N_{\delta_{k}}(F)}{-\ln\delta_{k}}$$

where *D_s_* is the fractal dimension, δ is the length of squares, *F* is the arbitrary non-empty bounded subset of space and $N_{\delta_{k}}$ is the number of $\delta_{k}$ grids intersecting *F*.

The box-counting method is also called the pixel-counting method because this method requires the use of 2D square lattices with different side lengths ($\delta_{k}$) to calculate the numbers ($N_{\delta_{k}}(F)$) of square lattices with different $\delta_{k}$ needed to cover *F*. Additionally, this method uses the least-squares approach to perform a unary linear regression on $\ln\delta_{k}$ and $\ln N_{\delta_{k}}(F)$. Thereby, the value of *Ds* is obtained. This method agrees with the fundamental definition of fractal theory and can be easily implemented by computer programming. Thus, the box-counting method is employed in this study to calculate the *D_s_* of the surface profiles of the workpieces.

## 3. Results and Discussion

### 3.1. Experimental Results

**Figure 3:**
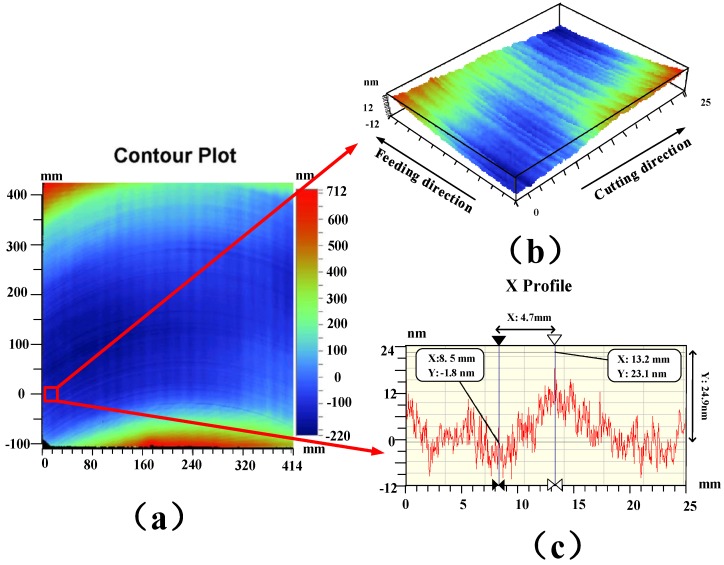
Machining surface results: (**a**) experimental results; (**b**) sample surface; (**c**) surface profile.

First, a KDP crystal plane with a diameter of 410 mm is machined on an ultra-precision fly-cutting lathe, with the machining parameters are shown in Table 1. This lathe is primarily used to machine large-diameter planar optical components and is capable of semifinished and finished machining of special hard and brittle materials such as KDP crystals. Combining the linear feed motion of the worktable with the rotation motion of the spindle, the single point diamond ultra-precision flying tool cutting of large-aperture optical planar elements can be realized. A WYKO NT9300 optical profiler (Veeco Instruments Inc., Plainview, NY, USA) is used to examine the form accuracy of the machined workpiece. Table 2 shows the measurement parameters of the measuring system. Figure 3 shows the experimental result. Figure 3a shows the results of the machined surface, and it can be seen that there is a significant texture on the surface. For further observation, a 25 mm-wide area outside the range of the cutting edge (the red area in Figure 3a) is extracted, as shown in Figure 3b. Figure 3c shows the 2D morphology of the surface in the cutting direction. As demonstrated in Figure 3b,c, notable waviness with a spatial period of 9.4 mm and an amplitude of approximately 24.9 nm is present on the machined surface along the cutting direction. The rotational speed (n) of the spindle is 300 rpm, and the radius (R) of the fly-cutter head is 315 mm. On this basis, the conversion space period to the time-frequency domain is about 526 Hz.

**Figure 4:**
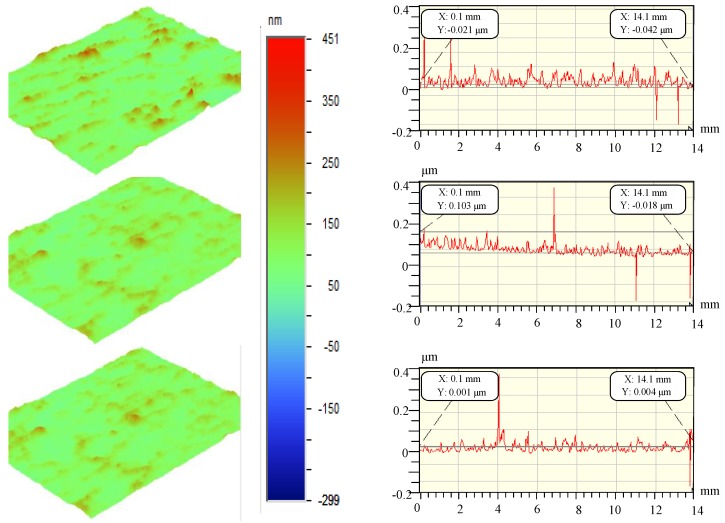
Al alloy surface profile.

**Figure 5:**
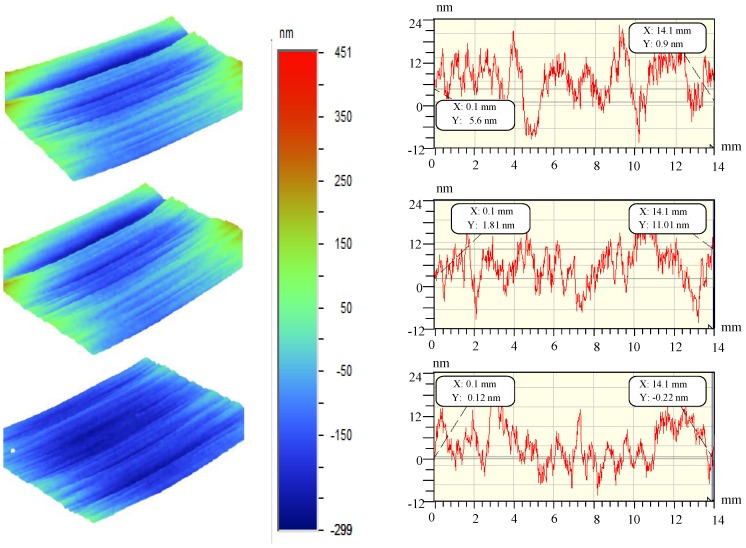
KDP crystal surface profile.

In order to study the directional characteristics of the surface of ultra-precision machining workpieces, ultra-precision fly-cutting machine tools are used to cut Al-alloy and KDP-crystal materials. During the machining process, the spindle speed is 200 rpm, the feed speed of the workbench is 30 µm/s, and the cutting depth is 4 µm. A total of six groups of data are measured. Due to the limited length of this article, sectional profiles are drawn based on the first three groups of data. Figure 4 and Figure 5 show the profiles of certain sections of the surfaces of the Al-alloy workpiece and the KDP-crystal workpiece, respectively.

### 3.2. Fractal Analysis of the Machined Surfaces

In order to study the differences in the morphology of the workpiece surfaces represented in Figure 4 and Figure 5, the three-dimensional fractal dimension *Ds* of the surface data for each workpiece is calculated in MATLAB (MATLAB R2016a, MathWorks, Natick, MA, America) using Equation (2) (i.e., the box-counting method), introduced in Section 2.4. Table 3 summarizes the *Ds* values. Based on the analysis of *Ds* in Section 2.4 and Table 3, the 3D *Ds* of the surface of the Al-alloy workpiece is mostly smaller than that of the surface of the KDP-crystal workpiece, which indicates that the surface of the KDP-crystal workpiece has a more complex microstructure.

Comparing the experimental results in Figure 4 and Figure 5 and the three-dimensional fractal dimensions in Table 3, it can be seen that the surfaces machined by fly cutting conform to the *D_s_* features. The finer a surface is, the higher the space-filling capacity of the surface is, and the higher the *D_s_* of the surface is. As demonstrated by the calculated data in Table 3, the *D_s_* associated with ultra-precision fly cutting generally ranged from 2.34 to 2.73, which indicates that the surfaces machined by fly cutting have a relatively complex morphological structure.

To validate the calculated values of *D_s_* in Table 3, a corresponding parameter *R_q_* (root-mean-square surface roughness) that characterizes the 3D-machined surface morphology was calculated. For discretely sampled data, *R_q_* can be calculated using the following equation,

(3) $$R_{q}=\sqrt{\frac{1}{l_{x}l_{y}}\int_{0}^{l_{y}}\int_{0}^{l_{x}}z^{2}(x,y)dxdy}=\sqrt{\frac{1}{MN}\sum_{j=1}^{N}\sum_{i=1}^{M}z^{2}(x_{i},y_{j})}$$

where, *z(x*, *y)* is the profile of surface, *l_x_* and *l_y_* are the side length of the sampling area and *M* and *N* are the number of discrete samples in the *x* and *y* direction within the sampling interval.

The measuring range of the WYKO NT9300 optical profiler on the two surfaces was 14 mm × 10 mm. There was a total of 640 × 480 sampling points. The *R_q_* of the machined workpieces in Figure 4 and Figure 5 was calculated using Equation (3). Table 4 summarizes the results. Based on Table 4, combined with the 3D *D_s_* of the machined surfaces in Table 1, the *R_q_* of the Al-alloy workpiece was higher than that of the KDP-crystal workpiece. However, the 3D *D_s_* of the Al-alloy workpiece was mostly lower than that of the KDP-crystal workpiece.

**Figure 6:**
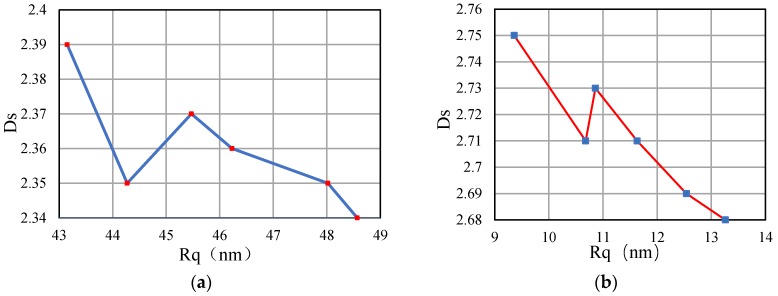
Relationship between surface roughness and fractal dimension: (**a**) Al alloy; (**b**) KDP crystal.

In order to analyze the relationship between the surface roughness of the workpiece and the three-dimensional fractal dimension, the *R_q_*–*D_s_* curves are plotted for the workpieces made of different materials based on the data in Table 3 and Table 4, as shown in Figure 6. As demonstrated in Figure 6, the *D_s_* of the surface of the workpiece basically decreases as *R_q_* increases. This result indicates that the *D_s_* values calculated in this study are accurate, thereby further demonstrating the theory that the greater the *D_s_* is, the finer the microscopic topology is. To further examine the relationship between *R_q_* and *D_s_*, a *D_s_* prediction model is established based on a backpropagation (BP) neural network.

### 3.3. Ds Prediction Model

**Figure 7:**
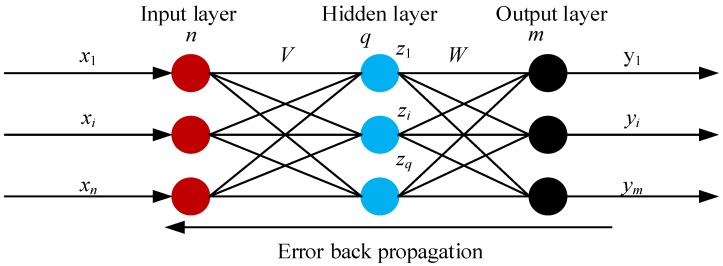
Network model.

A BP neural network is a multilayer feedforward neural network based on the error BP algorithm. A BP neural network describes the structure of a biological neural network with a certain simple mathematical model, and under certain algorithms, it can simulate the intelligent behavior of a biological neural network to some extent, so as to solve the problem of intelligent information processing which traditional algorithms cannot compete for. The network model is shown in Figure 7. Compared with traditional statistical methods, the BP neural network has the following advantages: it does not require the type or distribution of materials, it does not need to define the mathematical equation of the mapping relationship between input variables and output variables beforehand, and it can define the complex mapping relationship between them through self-learning and self-organization, so it has a good ability to deal with non-linear problems. Therefore, this paper chooses a BP neural network to establish the prediction model of the fractal dimension of the workpiece surface.

In order to improve the training accuracy of the BP neural network calculation, this paper normalizes all the data, and then carries on the inverse normalization after the training. The prediction model of the fractal dimension of the workpiece surface is established based on the BP neural network, and the modeling process is completed with the assist of the neural network toolbox of MATLAB. In this network, *R_q_* and *D_s_* are the input and output ends, respectively. The number of neurons in the hidden layer is calculated using an empirical equation.

(4) $$n=\sqrt{num\_input+num\_output}+a$$

Where, *n* is the number of neurons in the hidden layer, *num_input* is the number of input neurons, *num_output* is the number of output neurons and *a* is the constant between 1 and 10.

**Figure 8:**
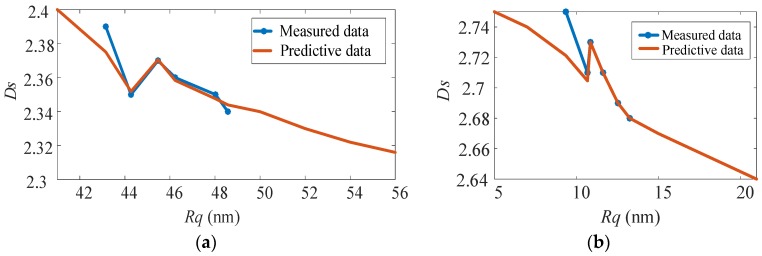
Prediction model of surface fractal dimension: (**a**) Al alloy; (**b**) KDP crystal.

Based on Equation (4) combined with the actual conditions, the network model established in this study has a relatively high prediction accuracy and convergence rate when there are eight neurons in the hidden layer. According to the fractal dimension of the surfaces of the machined workpieces in Figure 6, the prediction curves of the *R_q_* and *D_s_* of the surfaces of the machined workpieces are shown in Figure 8. Figure 8a shows the measured and predicted values of the *R_q_* and *D_s_* of the surface of the Al-alloy workpiece. Figure 8b shows the measured and predicted values of the *R_q_* and *D_s_* of the surface of the KDP-crystal workpiece. In Figure 8, the blue curves are based on the experimental data in Figure 6. The prediction model generated by training the network was capable of predicting the *D_s_* for other *R_q_* values, as shown by the red curves. The predicted curves were found to be very close to the measured experimental curves, demonstrating a very high prediction accuracy.

As demonstrated in Figure 8, the *R_q_* of each ultra-precision machined surface was essentially inversely proportion to the corresponding *Ds*. That is, the lower the surface roughness of the workpiece, the bigger its fractal dimension. However, there was no strict corresponding relationship between *D_s_* and *R_q_*. As demonstrated in Figure 8a, the *R_q_* of the Al-alloy workpiece ranged from 44 to 46, and *D_s_* was proportional to *R_q_*. This result suggests that there is no strict, direct relationship between the *R_q_* and *D_s_* of a workpiece; that is, *D_s_* is a surface characterization parameter that is independent of *R_q_*. In Figure 8a, the *R_q_* of the surface morphology of the Al alloy ranges from 42 to 44. The surface roughness is the same, but the fractal dimension is quite different. It can be further inferred that in the process of ultra-precision machining, the roughness of the machined surface can be kept unchanged by reasonable selection, while the surface microstructures change. This conclusion is of great significance for the study of optical elements. Therefore, our further research direction is to study the influence of cutting parameters on fractal features. It mainly analyzes the fractal characteristics of surface morphology, and the influence of various cutting parameters on surface roughness is not the focus of consideration. Only by fully understanding the influence of processing parameters on the fractal characteristics can we formulate reasonable processing parameters to optimize the surface quality of the workpiece and achieve the purpose of improving its optical performance.

### 3.4. Fractal Analysis of Surface Morphology Anisotropy

As mentioned in Section 2, similar to the physical properties of the material, the surface topology of a workpiece can be isotropic or anisotropic. When processing an anisotropic material, as the cutting direction of the tool changes with respect to the crystal orientation of the crystal, the deformation generated inside the crystal material also changes. When the material is plastically deformed, a smooth region is formed on the surface of the workpiece. When a brittle fracture occurs, a fracture failure zone is formed, which seriously affects the surface quality of the workpiece. The above phenomenon will cause anisotropy on the surface of the machined workpiece. Therefore, for the anisotropic materials, the *R_q_* and *D_s_* of the material vary in different directions. As a result, the statistical properties also vary in different directions. It can be seen from Figure 8 that even if the *R_q_* of a certain section of the workpiece surface morphology is the same, its *D_s_* may be different. Thus, to comprehensively analyze the surface information, the *D_s_* of the surface of each machined workpiece in various directions is analyzed.

**Figure 9:**
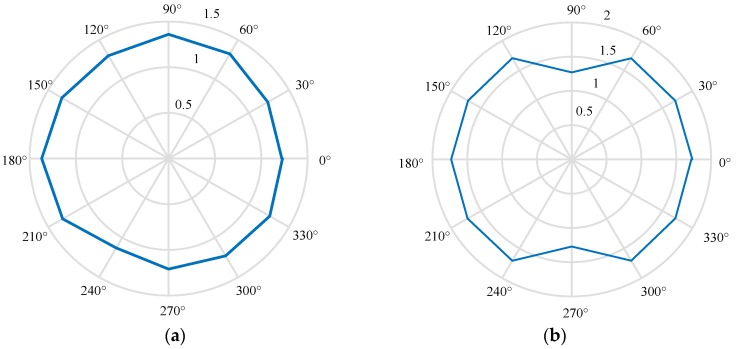
Fractal dimension of surface profile: (**a**) Al alloy; (**b**) KDP crystal.

Due to the limited length of this article, for the different materials, only a set of experimental data is selected to draw the *D_s_* of the surface profile in different directions, as shown in Figure 9. Figure 9b shows the *D_s_* of the surface profile of the KDP-crystal workpiece. The *D_s_* values of the surface profiles of this workpiece are mostly greater than 1.5, suggesting that the KDP-crystal workpiece has a relatively complex surface morphology. Further observation of Figure 9a,b shows that there is no significant fluctuation in the *D_s_* of the surface profile of the workpiece in Figure 9a in various directions, while the surface profiles of the workpiece in Figure 9b have a small fractal dimension of the surface contour in the 90° and 270° directions. As a result, the *D_s_* values of the circumferential profiles are more symmetrical in Figure 9b than in Figure 9a. The *D_s_* values in the 90° and 270° directions differ relatively significantly from those in other directions. This result also suggests that the profile features in the direction parallel to the 90° or 270° directions play a main role for the entire surface of the workpiece. Based on Thomas’ theory introduced in Section 2.2, the machined surface of this workpiece is anisotropic, indicating that the profiles parallel to the cutting direction are the main features of this surface.

## 4. 2D Power Spectrum Analysis

The power spectral density is established based on the analysis of the structure, so the power spectral density of the machined surface of the KDP crystal should be consistent with the fractal characteristics to a certain extent. To verify the fractal features of the surface of the KDP-crystal workpiece discussed in Section 3, the 2D PSD analysis method is employed. PSD can be derived from the fast Fourier transform (FFT) theory.

Performing a 2D FFT can be treated as processing two continuous one-dimensional (1D) FFTs. In practice, the surface of a workpiece is characterized based on a finite number of digital sampling points. Therefore, a 2D discrete FFT is often used for the numerical calculations in practice.

(5) $$G(\frac{u}{MT_{x}},\frac{v}{NT_{y}})=\sum_{q=0}^{N-1}\sum_{p=0}^{M-1}z(pT_{x},qT_{y})e^{-j2\pi \left|\frac{u}{M}p+\frac{v}{N}q\right|}$$

where *M* and *N* are the sampling points, and *T_x_* and *T_y_* are the sampling length.

In this study, the direct periodogram method is used to estimate the 2D PSD of the surface, and it is defined as follows:

(6) $$G(\frac{u}{MT_{x}},\frac{v}{NT_{y}})=\frac{1}{MNT_{x}T_{y}}{\left|H\left|\frac{u}{MT_{x}},\frac{v}{NT_{y}}\right|\right|}^{2}$$

where $\left|H\left|\frac{u}{MT_{x}},\frac{v}{NT_{y}}\right|\right|$ is the amplitude-frequency characteristics and $G(\frac{u}{MT_{x}},\frac{v}{NT_{y}})$, $\frac{1}{MNT_{x}T_{y}}$ is the sequence length.

**Figure 10:**
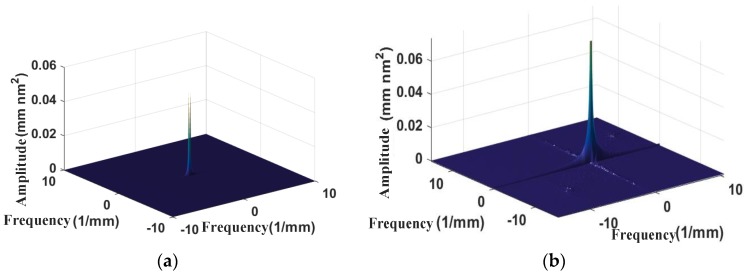
Surface power spectral density: (**a**) Al alloy; (**b**) KDP crystal.

When used to estimate spectra, the periodogram method can easily result in PSD energy leakage. Previous studies have shown that a data window can be used to effectively avoid this problem. Based on the above theory, a two-dimensional power spectral density analysis on the surface of KDP crystals and aluminum alloys processed by ultra-precision fly cutting is performed, as shown in Figure 10. Only one frequency-domain spectrum is present in the PSD diagram of the machined surface of the Al-alloy workpiece. The energy of the fly-cutting-machined surface of the KDP-crystal workpiece is mainly concentrated in the direction vertical to the surface texture. The machined surface is mainly affected by its spatial frequency in the direction vertical to the cutting direction. This result agrees with the finding obtained using the fractal method for 2D circumferential profiles—the features of the machined surface of the KDP-crystal workpiece consisted mainly of the profiles parallel to the cutting direction. Additionally, because the *D_s_* values of the profiles parallel to the cutting direction are relatively small, the PSD is insignificant in the direction parallel to the cutting direction. The analytical results for the PSD are consistent with those for the *Ds* of the circumferential surface profiles. This result demonstrates that fractal analysis can reflect not only the microstructure and anisotropy of a surface but also, to a certain extent, the spectral frequency distribution of the surface.

Based on the above analysis, the fly-cutting-machined surface of the KDP-crystal workpiece has a strong anisotropy, and its surface features are mainly composed of profiles in the cutting direction, which will cause waviness to a certain extent. Waviness can exert a significant impact on the surface quality of optical crystals. In severe cases, it may cause dispersion and severely affect the optical performance of optical-crystal components. Therefore, studying the cause of these waviness errors on machined surfaces is of great importance for improving the optical performance of crystals.

## 5. Workpiece Detection Signal Processing and Frequency Identification

The ultra-precision fly-cutting-machined surface of a KDP-crystal workpiece retains machining marks; that is, it has regular periodic components formed from feeding and random components resulting from various variables (e.g., spindle and ambient vibrations) and is a very complex mixed signal. The conventional *R_q_* is unable to represent the spatial frequency of the microstructure of such a surface. Especially for KDP crystals, special materials used in the field of nonlinear optics, the various frequencies on the surface will interfere with the characteristics of the laser beam at a specific wavelength. Being able to find a method to describe specific frequency characteristics, especially waviness characteristics, is of great significance for studying and evaluating the surface quality of crystal components. For a machined surface, the PSD method can be used to calculate the specific spatial frequency value contained in the surface, and a multiscale wavelet transform can be used to decompose the original surface on multiple scales.

Based on the above analysis combined with the results in Section 3 and Section 4, the features of the fly-cutting-machined surface of the KDP-crystal workpiece consisted mainly of the profiles in the cutting direction. In this study, wavelet and power spectral density methods are used to analyze the cutting direction profile of the machined surface of the KDP crystals in the frequency domain, which lays a theoretical foundation for analyzing the frequency characteristics of the surface microtopography, especially the relationship between the waviness characteristics and various processing factors.

**Figure 11:**
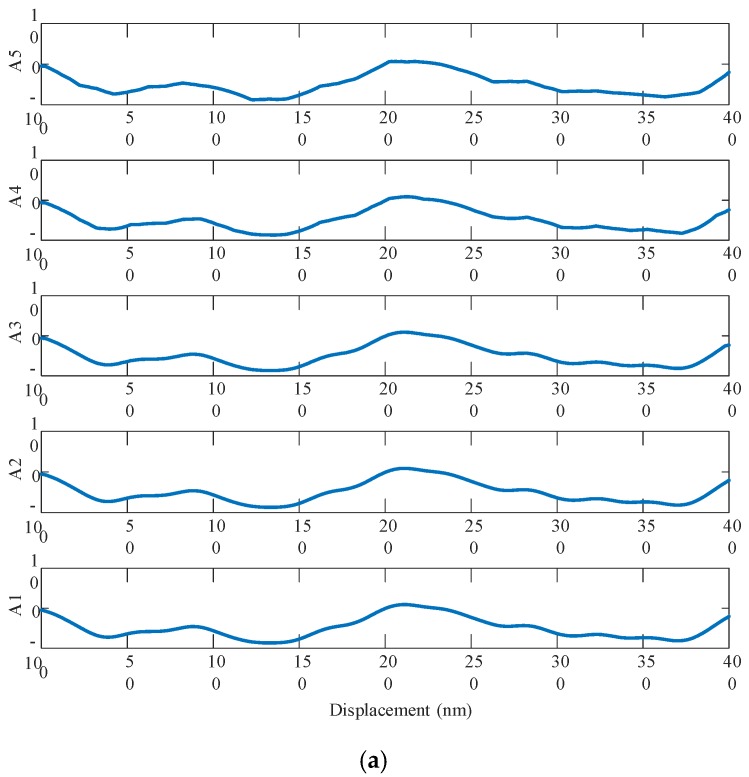

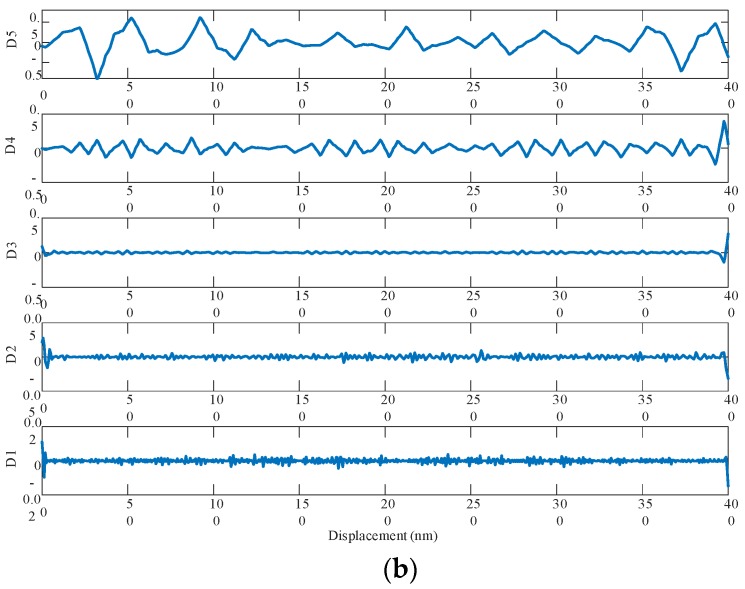
Wavelet transform decomposition result: (**a**) Low-frequency signal; (**b**) High-frequency signal.

A 1D discrete wavelet transform is performed to analyze the profiles of the machined surface of the KDP-crystal workpiece in Figure 3. Prior to analysis, the signals are filtered to eliminate the effects of the test instruments. The compactly supported orthogonal wavelet base db3 (Third-level Daubechies wavelet) is used to perform five-level decomposition and analysis on the surface frequency information for the KDP-crystal workpiece. Figure 11 shows the reconstructed low-frequency (A1–A5) and high-frequency (D1–D5) signals. As demonstrated in Figure 11a, each low-frequency surface signal is a combination of multiple frequency features and exhibits a topology similar to the original surface profile. Figure 11b shows the high-frequency surface information obtained from wavelet decomposition. As demonstrated in Figure 11b, D4 and D5 have relatively large form errors, corresponding to the waves visible on the surface. However, the surface-frequency information obtained from the wavelet method is limited. Therefore, the PSD method is used to further analyze the surface.

**Figure 12:**
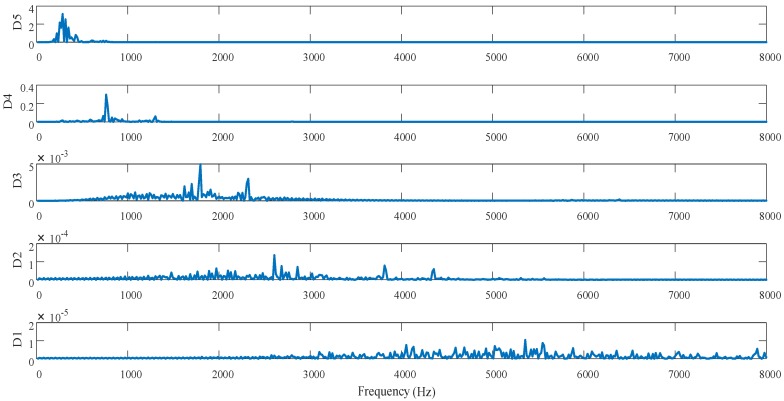
Power spectrum of high-frequency signal after wavelet decomposition.

The PSD method is used to analyze the PSD of the high-frequency information obtained from wavelet decomposition in Figure 11, as shown in Figure 12. To directly identify the relationship between the surface profile and vibration, Equation (7) is used to transform the high-frequency information obtained by wavelet decomposition for the surface profiles from the spatial domain to the frequency domain [6]:

(7) $$\omega_{n}=\frac{\pi R\times n\times \cos\theta }{60T_{D}}$$

where *θ* is the maximum angle of the tangential direction during cutting and *θ* = 0°, *T_D_* is the spatial period of surface waviness and *n* is the frequency of surface waviness.

**Figure 13:**
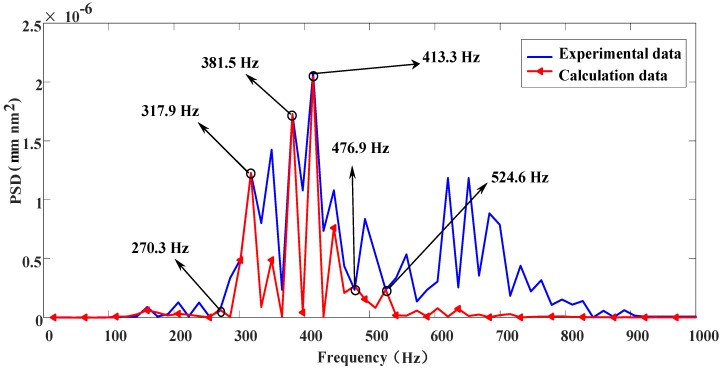
Comparison of PSD between simulation and experimental results.

It can be seen from Figure 12 that the corresponding frequency range is different on each scale, and the variation of the high-frequency PSD on each scale is caused by a specific reason. For the machined surface studied, the frequency components of D1–D4 are mainly related to the material properties and anisotropic cutting parameters and corresponded to relatively high frequency after wavelet decomposition. The frequency components of D5 are primarily related to the spindle vibration and were relatively low. To further identify the vibration frequency caused by spindle vibration, the simulated displacement and the PSD of the detail signal D5 in Figure 12 are comparatively analyzed, as shown in Figure 13. As demonstrated in Figure 13, the relatively high-frequency waves (frequency: 270–600 Hz) on the machined surface of the optical KDP-crystal workpiece are caused by spindle vibration.

## 6. Conclusions

In this study, wavelet, fractal, and PSD methods are employed to analyze the machined surface of a KDP-crystal workpiece. Additionally, the relationship between the surface roughness and the fractal dimension during the ultra-precision fly-cutting process is examined. Moreover, the anisotropy of the surface morphology is analyzed. Furthermore, based on the orientation of the machined surface, the cause of surface waviness errors is revealed. The main conclusions derived from this study are summarized as follows:

(1) The fractal and 2D PSD methods are used to calculate and analyze the features of the machined surfaces of various materials. The machined surface of the KDP-crystal workpiece exhibits strong anisotropy, and the features of the surface are mainly composed of contours in the cutting directions.
(2) The wavelet-transform and PSD methods are employed to analyze the tangential waves on the machined surfaces and identify the scale of the anisotropy and spindle vibration for various materials from a frequency-domain perspective. Additionally, the frequency range of the spindle vibration is identified based on the machining experiment. On this basis, the cause of machined surface waviness errors is revealed.

## Figures and Tables

**Table 1:** Parameters for surface machining.

Parameters	Values
Spindle speed *n* (rpm)	300
Feed rate *f* (μm/s)	30
Cutting depth *a_p_* (μm)	4
Tool rake angle (°)	45
Tool radial error (xS (μm))	10
Tool error angle β (°)	0.5
Radius of cutter head *R* (mm)	315
Workpiece width *W* (mm)	410

**Table 2:** Measurement parameters of the measuring system.

Parameters	Values
Sampling interval	0.01 ms
Roughness RMS repeatability	0.005 nm
Measuring range	14 mm × 10 mm
Kind of filter	remove shape standard filtering filter spectrum
Z-direction resolution	0.1 nm

**Table 3:** Fractal dimension (*Ds*) of the workpiece surface.

Material	*D_s_*					
	1	2	3	4	5	6
Al alloy	2.37	2.35	2.34	2.35	2.39	2.36
KDP crystal	2.71	2.75	2.68	2.73	2.69	2.71

**Table 4:** Workpiece surface roughness (*R_q_*).

Material	*R_q_*					
	1	2	3	4	5	6
Al alloy	45.47 nm	48.02 nm	48.57 nm	44.27 nm	43.15 nm	46.23 nm
KDP crystal	10.68 nm	9.36 nm	13.26 nm	10.86 nm	12.54 nm	11.63 nm
